# Strategies to Detect Hepatic Myofibroblasts in Liver Cirrhosis of Different Etiologies

**DOI:** 10.1007/s40139-014-0057-8

**Published:** 2014-09-14

**Authors:** Keiko Iwaisako, Kojiro Taura, Yukinori Koyama, Kenji Takemoto, Masataka Asagiri

**Affiliations:** 1 Department of Target Therapy Oncology, Kyoto University Graduate School of Medicine, 54 Kawaharacho, Shogoin, Sakyo-Ku, Kyoto, 606-8507 Japan; 2 Division of Hepato-Biliary-Pancreatic and Transplant Surgery, Department of Surgery, Kyoto University Graduate School of Medicine, Kyoto, 606-8507 Japan; 3 Innovation Center for Immunoregulation and Therapeutics, Graduate School of Medicine, Kyoto University, Kyoto, 606-8507 Japan

**Keywords:** Liver cirrhosis, Hepatic fibrosis, Myofibroblasts, Hepatic stellate cells, Portal fibroblasts

## Abstract

Liver cirrhosis, a late stage of hepatic fibrosis, is an increasing cause of morbidity and mortality worldwide. Hepatic fibrosis is mainly caused by alcoholic or non-alcoholic steatohepatitis, chronic viral hepatitis, or autoimmune and biliary diseases. Myofibroblasts, which are absent from the normal liver, are differentiated from heterogeneous cell populations in response to a liver injury of any etiology and produce the extracellular matrix. Hepatic stellate cells are considered the main source of myofibroblasts. However, the origin of hepatic myofibroblasts remains unresolved, and despite considerable research, only a limited success has been achieved by existing anti-fibrotic therapies. The question remains whether these limitations are caused by lack of attention to the critical targets, the myofibroblasts derived from cells of other mesenchymal origins. Therefore, identifying the origin of myofibroblasts may provide insight into the mechanisms underlying liver fibrosis, and may lead to the development of more effective therapies. This review will examine our current strategies for detecting hepatic myofibroblasts of different origins.

## Introduction

Liver cirrhosis (LC) is a major, life-threatening health problem worldwide. LC results from liver injuries of numerous different etiologies, causing hepatocyte damage, hepatic inflammation, and fibrogenesis [1]. LC can lead to the development of hepatocellular carcinoma [2]. LC is histologically characterized by increased deposition in and altered composition of the extracellular matrix (ECM) and the appearance of regenerative nodules. The destruction of the normal architecture of the liver and the loss of its functional hepatocytes prevent the liver from performing its normal detoxification, synthesis, and metabolic functions, eventually leading to portal hypertension and liver failure. From a clinical standpoint, LC is regarded as an end stage disease that leads to death, unless a liver transplant is performed [3]. However, several problems are associated with liver transplantation, such as a shortage of donors, post-transplant rejection, operative risk, and high costs.

Recently, it has become increasingly clear that hepatic fibrosis is reversible if its causative agents are successfully targeted; this has proved to be the most effective treatment for LC thus far [4]. However, the underlying causative agents are treatable only in subsets of patients with liver disease. Although there has been considerable research on liver fibrosis, there are no specific treatments for this condition. An ideal anti-fibrosis therapy would be specific for fibrogenic cells in the liver and be effective in attenuating excessive ECM deposition.

Myofibroblasts are the main effector cells in the fibrotic liver. In both experimental and clinical liver fibrosis cases, myofibroblasts appear and produce ECM at the site of the hepatic injury. The activation of ordinarily quiescent hepatic stellate cells (HSCs) into myofibroblasts is considered a major pathway of hepatic fibrogenesis associated with liver injury and has thus dominated the focus of studies on liver fibrosis [5]. The activated HSCs or their resulting myofibroblasts were the first major cell type in the liver to be identified as prominent in producing ECM in the injured liver [6]. Currently, at least three sources of myofibroblasts in liver fibrosis have been proposed. The hepatic resident mesenchymal cells [7], consisting of the quiescent HSCs and the portal fibroblasts, can differentiate into myofibroblasts. Then, bone-marrow derived cells, consisting of fibrocytes and mesenchymal stem cells in the peripheral blood, can be recruited to the injured liver to differentiate into myofibroblasts. Recent studies have demonstrated that bone-marrow derived cells make only a small contribution to the myofibroblast population in experimental liver fibrosis. Instead, fibrocytes may play a crucial role in the initiation of immune response during the earliest phases of tissue injury [8•]. Finally, hepatic progenitor cells, hepatocytes, cholangiocytes, and hepatic sinusoidal endothelial cells have been proposed to differentiate into myofibroblasts through epithelial or endothelial mesenchymal transition (EMT). Although primary hepatocytes can undergo EMT in vitro, it is extremely hard to detect hepatic myofibroblasts originating from epithelial or endothelial cells through EMT in vivo [9]. Thus, the main sources of hepatic myofibroblasts in liver fibrosis are the hepatic resident mesenchymal cells, consisting of the HSCs and portal fibroblasts. The most widely used and accessible marker of myofibroblasts are the de novo expression of α-smooth muscle actin (α-SMA). However, no reliable markers have yet been identified for distinguishing HSCs from portal fibroblasts after myofibroblastic differentiation. The contribution of portal fibroblasts to hepatic fibrosis is not well understood mainly because of the difficulties in distinguishing and isolating them.

Development of liver fibrosis and cirrhosis is associated with deposition of ECM, in which Collagen Type I is the most abundant [10]. Transgenic Colagen-α1(I)-GFP mice have been generated a decade ago. In these mice expression of GFP is driven by Collagen-α1(I) promoter, and therefore, expression of GFP is observed in cells that upregulate Collagen Type I. Our current review will summarize the recent results obtained from transgenic reporter mice and novel flow cytometry protocols developed to distinguish HSC- and portal fibroblast-derived myofibroblasts and quantify their relative contributions to hepatic fibrosis.

## Fibrotic Cascade in the Liver

Once hepatic epithelial cells (hepatocytes and/or cholangiocytes) are damaged by any cause, inflammatory mediators are released to initiate a series of responses to liver injury. Inflammatory cells recruited to the site of injury phagocytose necrotic or apoptotic cells and amplify the inflammatory response by releasing pro-inflammatory cytokines, such as tumor necrosis factor-α (TNF-α), interleukin-6 (IL-6), and interleukin-1 beta (IL-1β), and by recruiting T cells [11]. The hepatic mesenchymal precursor cells of myofibroblasts are activated and differentiated by growth factors and cytokines including transforming growth factor-beta (TGF-β), platelet-derived growth factor (PDGF), and interleukin-13 (IL-13). TGF-β drives myofibroblast activation and ECM synthesis. PDGF stimulates HSC proliferation through its positive feedback mechanism involved in the autocrine and paracrine effect. IL-13 has been also implicated in stimulation of TGF-β synthesis in cells [12].

Immune cells play a pivotal role in the development of hepatic fibrosis. In experimentally induced fibrosis, the balance between Th1 and Th2 cells is important for the fibrotic response. For example, C57BL/6 mice (in which a Th1 cell response predominates) have a lower fibrotic reaction than BALB/c mice (in which a Th2 cell response predominates) [13]. Recently, increasing evidence has suggested an emerging novel role of T cell subsets, including Th17, Treg, and δγT cells, in the fibrotic process [14]. However, further studies are needed to fully elucidate their functions.

Hepatic fibrosis is a dynamic process and can be considered a part of the healing response to liver injury. The ECM is not stable, but is constantly synthesized and degraded by proteolytic enzymes such as the matrix metalloproteinases (MMP) or collagenases. The reversibility of mild to moderate hepatic fibrosis is now a reality in patients whose etiology has been successfully treated. In clinical practice, studies of antiviral treatments for hepatitis C have showed that fibrosis is reversible after a sustained virologic response [15]. However, there is no unequivocal evidence for a complete reversal of severe cirrhosis with regenerative nodules and dense fibrotic septa. Current clinical studies based on liver biopsies have showed that the matrix enzyme lysyl oxidase–like-2 (LOXL2) increased in the fibrotic liver and was limited in the healthy liver. LOXL2 catalyzes the first step in the formation of crosslinks in fibrillar collagen. The extensiveness of the crosslinks observed in severe fibrosis and cirrhosis prevent their degradation by collagenases [16].

## Hepatic Myofibroblasts

Hepatic myofibroblasts, characterized by expression of α-SMA and production of ECM, are mainly found in chronically injured livers, irrespective of the etiology, and are morphologically defined as large and spindle-shaped cells with cytoplasmic stress fibers running parallel to the long axis. Myofibroblasts are characterized by several common features based on their ultrastructural analysis, including a prominent rough endoplasmic reticulum, a Golgi apparatus producing collagen, peripheral myofilaments, well-developed cell-to-stroma attachment sites (fibronexus), and gap junctions [17, 18]. The process of myofibroblast differentiation leads to a highly proliferative, migratory, and contractile phenotype. The persisting inflammation is believed to drive and sustain fibrogenesis. Myofibroblasts can release a number of pro-inflammatory molecules and directly contribute to this continuous inflammation [10, 19, 20]. In both experimental and clinical liver fibrosis, there is a close correlation between the regression of liver fibrosis and the disappearance of myofibroblasts. Previous studies have demonstrated that some myofibroblasts undergo cell death by apoptosis, while other myofibroblasts are restored to their quiescent-like state [21•, 22]. This phenomenon has been identified recently, but has a great potential for anti-fibrotic therapy. However, the mechanism underlying “inactivation” of HSC/myofibroblasts in response to toxic liver injury remains unknown. Future investigations are required to determine why a half of HSC/myofibroblasts apoptose during regression of liver fibrosis, while the other half of myofibroblasts survives and undergoes inactivation. Identification of the mechanism of HSC/myofibroblast inactivation, may provide new targets for anti-fibrotic therapy.

## Two Experimental Models for the Study of Hepatic Fibrosis

Mouse models have been used for several decades to study fibrogenesis. The two most common methods for modeling experimental liver fibrosis in mice are the administration of carbon tetrachloride (CCl_4_) and bile duct ligation (BDL). Each model displays specific characteristics in the evolution of fibrosis.

Administration of CCl_4_ leads to centrilobular necrosis, and eventually leads to liver fibrosis and cirrhosis. CCl_4_ causes damage of hepatocytes, in which highly reactive free radical metabolites are formed by the mixed function oxidase system, including a CYP2E1-mediated reaction [23]. HSCs are activated following CCl_4_ challenges. In this model, fibrosis first develops in pericentral areas and secondarily between central and portal areas, which is called “bridging fibrosis.”

The hepatic injury induced by BDL in mice is similar to the condition of human secondary biliary cirrhosis; it is characterized by cholestasis, hepatic inflammation, neutrophil infiltration in the portal tracts, proliferation of cholangiocytes, and portal tract fibrosis. In BDL mice, serum bile acid levels increase by dozens of fold. Bile acids are pro-oxidants directly causing tissue damage mediated by reactive oxygen species (ROS), or indirectly through activation of Kupffer cells to release ROS [24]. The overspill of bile acid stimulates the proliferation of cholangiocytes, resulting in a ductular reaction accompanied by portal inflammation and fibrosis [25•]. Previous studies have showed the importance of portal fibroblasts as contributors to fibrosis in the BDL model [26].

## Hepatic Stellate Cells (HSCs)

HSCs are intralobular connective tissue cells representing less than ten percent of the total number of liver cells. Under physiological conditions, HSCs reside in the space of Disse and serve as a major storage of Vitamin A in the mammalian body. HSCs also participate in the homeostasis of the intrahepatic ECM protein turnover by secreting the sufficient amount of ECM molecules required for tissue repair and by releasing MMP and their inhibitors. By virtue of the contractility of their long cytoplasmic processes encircling the sinusoid, HSCs presumably contribute to the regulation of hepatic microcirculation through the sinusoidal capillaries [27].

The liver is the main storage organ for dietary Vitamin A. Vitamin A includes numerous retinoid forms such as retinyl esters, retinol, retinal, retinoic acid, and several provitamin A carotenoids. Retinoids are transported in the form of retinyl esters. Dietary retinoids are absorbed in the small intestine, where they are packaged into chylomicrons for transportation to the lymphatic circulation system. The retinoid-containing chylomicrons are taken up by hepatocytes, wherein retinoids are hydrolyzed to retinol, and bound retinol-binding protein (RBP), to transfer to the HSCs for storage. HSCs are the central cellular site for retinoid storage in healthy animals, accounting for as much as 50–60 % of the total retinoid present in the entire body. Retinoids are stored in the form of retinyl esters in the lipid droplets, which are characteristic of HSCs [28]. In response to liver injury, quiescent HSCs activate and release some of the Vitamin A droplets. Upon activation, HSCs change their morphology, migrate to the site of injury, and upregulate mesenchymal markers such as α-SMA, collagen α1(I), and fibronectin. HSCs differentiate into myofibroblasts in the injured liver and produce ECM [29].

## Portal fibroblasts

Portal fibroblasts are resident fibroblasts with a spindle shape which are present in very small numbers in the mesenchyme surrounding the bile ducts. Under normal conditions, they participate in physiological ECM turnover. Portal fibroblasts almost certainly give rise to myofibroblasts during the development of cholestatic liver injury (but not toxic liver injury, [30]). In response to hepatic injury induced by BDL in mice, portal fibroblasts proliferate and are activate to produce ECM at the periphery of the bile ducts [31]. Portal fibroblasts can be distinguished from HSCs due to the lack of oil droplets, including Vitamin A. In addition, they express elastin and Thy-1; elastin, fibulin 2, gremlin 1, and mesothelin (a novel marker) have also been identified as markers of portal fibroblasts [32, 33]. However, during the development of hepatic injury, HSCs slightly express elastin [34]. Thy1 is a T cell marker, which is particularly abundant on the surface of thymocytes and peripheral T cells. Therefore, the question is, what are the specific markers for portal fibroblasts, and how portal fibroblasts can be distinguished from other myofibroblasts in fibrotic liver. In chronic cholestatic disorders, the fibrotic tissue is initially located around portal tracts. Histological findings from fibrotic livers suggested that portal fibroblasts contribute to the overall fibroblasts observed in cholestatic liver injury. However, their role in liver fibrosis is still unclear because of the lack of markers that can definitively determine the presence of portal fibroblasts from the pool of hepatic myofibroblasts. This problem is further complicated by a recent report by Asahina et al., suggesting that portal fibroblats and HSCs may originate from a common progenitor during the embryonic development [7].

## Strategies to Detect Hepatic Myofibroblasts

In recent years, manipulation of mouse genetics has been remarkably progressed and provided tools that have greatly facilitated the studies designed to dissect many biological processes in mammalian body, including liver fibrosis. Thus, development of collagen-α1(I)-GFP mice became one of the useful tools to study liver fibrosis [9, 17, 21•, 35••]. Our group has also utilized the collagen-α1(I)-GFP transgenic mouse in which green fluorescent protein (GFP) is upregulated in hepatic myofibroblasts in response to fibrogenic liver injury [36]. These mice can undergo chronic liver injury with repeated CCl_4_ injections or BDL to induce liver fibrosis, after which their collagen-producing cells express GFP, which is easily identified by its GFP fluorescence. The expression of collagen-α1(I)-driven GFP in these mice closely correlates with the expression of α-SMA, a general marker for myofibroblasts. The GFP-expressing cells have been considered myofibroblasts [35]. Our strategy to detect hepatic myofibroblasts was based on the investigation of GFP-expressing cells in nonparenchymal fractions of CCl_4_-treated or BDL collagen-α1(I)-GFP mice.

The study of the cell fate mapping of HSCs had demonstrated that although there is a decrease in the amount of Vitamin A upon HSC activation, the Vitamin A-specific autofluorescence excited with UV can be still detected in all HSCs by flow cytometry [35, 37]. Whereas the GFP is expressed in all myofibroblasts, the presence of droplets containing of Vitamin A is solely and exclusively attributed to HSC-derived myofibroblasts [35, 37, 38]. To distinguish HSCs from hepatic myofibroblasts of other origins, the flow cytometry has been reported to be a method of choice to distinguish and quantify the contribution of HSCs and portal fibroblasts to liver fibrosis induced by either CCl_4_ treatment or BDL. The suggested method used GFP to identify all myofibroblasts. Next, the presence of Vitamin A was used to identify myofibroblasts originated from HSCs, while all other GFP + Vitamin A- myofibroblasts were attributed to myofibroblasts of all other origins. Surprisingly, this GFP + Vitamin A- fraction was composed mostly by Thy1 and TE-1 (elastin) positive cells, while CD45 + Collagen-α1(I)-GFP + fibrocytes [39•] constituted only 4 % of total GFP + fraction. Taken together, the flow cytometry-based quantification analysis of hepatic myofibroblasts activated in fibrotic liver in response to different types of liver injury (toxic and cholestatic) has demonstrated that HSCs are the major source of myofibroblasts in CCl_4_-incuced liver fibrosis. However, portal fibroblasts are the major source of myofibroblasts at the onset of BDL-induced liver injury, within a week of BDL. The relative contribution of portal fibroblasts decreases upon chronic cholestatic injury, as HSCs become progressively activated and contribute to the myofibroblast population. Remarkably, the phenotype of BDL-activated HSCs has more similarities with BDL-activated portal fibroblasts rather than with CCl_4_-activated HSCs, suggesting that portal fibroblasts might affect (or even regulate) activation of HSCs in BDL-injured liver.

**Fig. 1:**
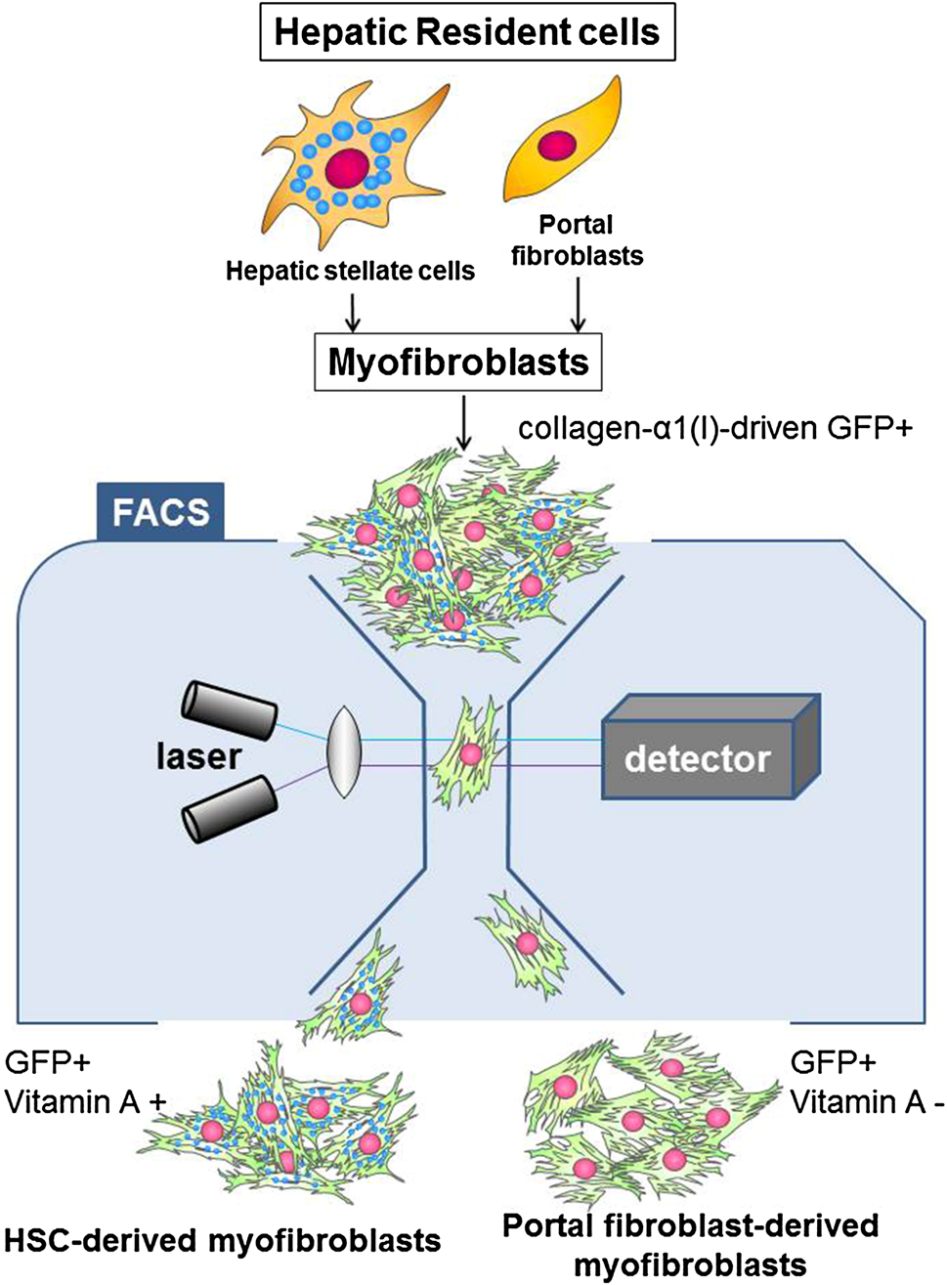
Strategy to analysis myofibroblasts by flow cytometry: Myofibroblasts expressing collagen-α1(I)-driven GFP+ are identified in nonparenchymal fraction by argon laser at 488 nm wavelength and further fractionated to Vitamin A+ and Vitamin A− cells by UV laser. HSC-derived myofibroblasts are sort-purified as the GFP+ and Vitamin A+ fraction. Portal fibroblast-derived myofibroblasts are sort-purified as the GFP+ and Vitamin A− fraction

This observation was supported by the gene expression array. Both of these cellular populations were isolated from mouse liver by flow cytometry and the gene expression profile was determined for GFP + Vitamin A + and GFP + Vitamin A- populations from CCl_4_- and BDL-injured mice. Gene expression profiling and complimentary immunohistochemistry revealed that myofibroblasts derived from HSCs are positive for desmin, and myofibroblasts derived from portal fibroblasts express Thy1, elastin, and mesothelin [35]. Mesothelin is a membrane glycoprotein that is expressed in normal mesothelial cells; however, its function is not clear. In our study, mesothelin was highly expressed in myofibroblasts derived from portal fibroblasts, such that mesothelin may serve as a novel marker of portal fibroblasts. Despite this finding, the function of mesothelin in mice or humans is not yet clear. In addition, recent studies have suggested that liver capsule (which may also express mesothelial markers) can contribute to hepatic myofibroblasts in response to fibrogenic liver injury [7, 40]. At this time, it remains unclear if the mesothelin+ myofibroblasts represent heterogeneous population of hepatic mesenchymal cells that emerge in the damaged liver in response to chronic injury, or is comprised by the same cell type at different stages of activation. Taken together, there might be two major sources of hepatic myofibroblasts in fibrotic liver [39•]. These populations of myofibroblasts may behave similar to each other (Fig. 1), but they exhibit unique properties, and can be distinguished from each other based on their gene expression profile. Therefore, we emphasize that the composition of myofibroblasts varies depending on the etiology of the hepatic injury, and the origin of myofibroblasts may determine the personalized anti-fibrotic therapy of patients with liver fibrosis of different etiologies [35••, 41].

## Conclusions

Myofibroblasts are the source of the fibrous scar tissue in liver fibrosis. Hepatic myofibroblasts are transdifferentiated from two main cell populations in response to hepatic injury. The major origins of hepatic myofibroblasts are HSCs and portal fibroblasts. Fibrocytes also contribute to liver fibrosis but their function is not well characterized. Liver fibrosis caused by hepatotoxic injury is attributed to the activated HSCs. However, portal fibroblasts are implicated in liver fibrosis induced by cholestatic liver injury. The contribution of portal fibroblasts to liver fibrosis has not been well characterized because of the difficulties in cell sorting-purification and the lack of identifiable and specific markers for portal fibroblasts. Our novel flow cytometry method makes it possible to distinguish HSC- and portal fibroblast-derived myofibroblasts from the nonparenchymal cell fraction of the fibrotic liver in mice. It is also able to identify a novel specific marker, mesothelin, which is specific to portal fibroblasts. A detailed investigation of myofibroblasts, particularly using new methods such as ours, will provide insight into the mechanisms underlying liver fibrosis, and may lead to the development of more effective therapy.
